# The Role of Reactive Species in Epileptogenesis and Influence of Antiepileptic Drug Therapy on Oxidative Stress

**DOI:** 10.2174/157015912804143504

**Published:** 2012-12

**Authors:** Boštjan Martinc, Iztok Grabnar, Tomaž Vovk

**Affiliations:** Faculty of Pharmacy, University of Ljubljana, Aškerčeva 7, 1000 Ljubljana, Slovenia

**Keywords:** Antiepileptic drugs, antioxidants, calcium, epileptogenesis, mitochondria, oxidative stress, reactive species, signalling.

## Abstract

Epilepsy is considered one of the most common neurological disorders. The focus of this review is the acquired form of epilepsy, with the development process consisting of three major phases, the acute injury phase, the latency epileptogenesis phase, and the phase of spontaneous recurrent seizures. Nowadays, an increasing attention is paid to the possible interrelationship between oxidative stress resulting in disturbance of physiological signalling roles of calcium and free radicals in neuronal cells and mitochondrial dysfunction, cell damage, and epilepsy. The positive stimulation of mitochondrial calcium signals by reactive oxygen species and increased reactive oxygen species generation resulting from increased mitochondrial calcium can lead to a positive feedback loop. We propose that calcium can pose both, physiological and pathological effects of mitochondrial function, which can lead in neuronal cell death and consequent epileptic seizures. Various antiepileptic drugs may impair the endogenous antioxidative ability to prevent oxidative stress. Therefore, some antiepileptic drugs, especially from the older generation, may trigger oxygen-dependent tissue injury. The prooxidative effects of these antiepileptic drugs might lead to enhancement of seizure activity, resulting in loss of their efficacy or apparent functional tolerance and undesired adverse effects. Additionally, various reactive metabolites of antiepileptic drugs are capable of covalent binding to macromolecules which may lead to deterioration of the epileptic seizures and systemic toxicity. Since neuronal loss seems to be one of the major neurobiological abnormalities in the epileptic brain, the ability of antioxidants to attenuate seizure generation and the accompanying changes in oxidative burden, further support an important role of antioxidants as having a putative antiepileptic potential.

## INTRODUCTION

Epilepsy is one of the most common and heterogeneous neurological disorders, with an estimated prevalence of 40 to 50 million patients worldwide [1]. The incidence of epilepsy is particularly high in children under 5 years of age and in individuals older than 65 years [2, 3]. About one fourth of all cases develop before the age of five [4]. 

Epilepsy or epileptic condition is defined by a state of recurrent, spontaneous (unprovoked) seizures, which can be convulsive or non-convulsive episodes. It is characterized by synchronized abnormal electrical activity arising from a group of cerebral neurons [3, 5, 6].

Nowadays, the increasing attention is paid to the possible connection between oxidative stress that results in disturbance of physiological signalling roles of Ca^2+^ which is associated with the increased production of free radicals, mitochondrial dysfunction, cell damage and consequently epilepsy.

Mitochondria are well known as the main source of the cell's adenosine triphosphate (ATP). In numerous cell types they also serve as Ca^2+^ buffer. As a consequence of Ca^2+ ^influenced ATP production in the mitochondria, reactive oxygen species (ROS) are generated. It is supposed that Ca^2+^ ions, as well as ROS, under physiological conditions work as positive effectors and cellular signalling molecules within mitochondrial signalling pathways, while their overload can lead to mitochondrial dysfunction, oxidative stress and cell damage. Neuronal dysfunction or death could serve as the propagating factor that may lead to the epileptic condition.

This review is focused on acquired epilepsy and comprehensively examines the evidence for the initial role of subtle disturbance in Ca^2+^ and ROS cellular balance in epileptogenesis. Furthermore, the influence of anticonvulsant therapy on endogenous ability to prevent oxidative stress will be reviewed. 

## ETIOLOGY OF EPILEPSY

Epilepsy can be either idiopathic or symptomatic. It is estimated that up to 50% of epilepsy cases are symptomatic or acquired, therefore associated with a previous neurological insult [7]. Acquired form of epilepsy does not have a genetic link and becomes more common as people age. It is more widespread than genetic or idiopathic form. Idiopathic epilepsy is mainly due to genetic causes or developmental central nervous system (CNS) disorders and malformations, that most often affect mitochondrial or ion channel function. There are many genetic factors that contribute to the increased neuronal excitability present in most idiopathic epilepsies. Any mutation or defect in mitochondrial respiratory chain complexes, synapses, and neurotransmitter receptors or in the voltage and ligand channels may alter brain excitability and cause epileptic seizures. Epilepsy is in most cases multifactorial. It often arises in part from both, the genetic and the acquired factors. Aetiology varies among various age groups, patient groups and geographical locations. In general, congenital and perinatal conditions are the most common causes of early childhood epilepsy onset; while in adults, epilepsy is more likely to be due to external non-genetic causes. However, this distinction is by no means absolute. 

The epilepsies are normally divided into ‘early’ (i.e. seizures occurring within a week of the insult) and ‘late’ (i.e. chronic epilepsy developing later). Between the injury and the onset of late epilepsy, there is normally a ‘latent period’. During this period, most likely epileptogenic processes are developing, which raises the possibility for neuroprotective interventions to prevent later epilepsy. 

## THE DEVELOPMENT PHASES OF SYMPTOMATIC EPILEPSY

It is generally accepted that development processes of acquired epilepsy occur in three major phases: the acute phase - injury, the latency phase - epileptogenesis, and finally the phase of spontaneous recurrent seizures - chronic epilepsy [8]. 

### The Acute Phase – Injury

Epileptogenesis in acquired epilepsy can be initiated by a number of types of brain lesions and these numerous aetiologies may vary with age. Illnesses in the form of tumours, infections, status epilepticus, childhood febrile seizures, stroke, hypoxia and neurodegenerative diseases, increase the incidence of acquired epilepsy. Furthermore, status epilepticus, stroke, and traumatic brain injury are considered as the three major causes of brain injuries and acquired epilepsy [7]. 

Sometimes CNS insults are associated with immediate seizures in the acute setting, but since epileptogenesis phase is required, this does not necessarily mean that it will lead to acquired epilepsy [8].

### The Latent Phase - Epileptogenesis

The process whereby an initial central nervous system insult leads to the development of the epileptic condition is referred to as epileptogenesis. This transition processes may take years or even decades after the initial insult. This might suggest that a progression of changes in response to the initial insult is critical to the development of the epileptic condition [3, 9]. Furthermore, epilepsy is usually not a static condition, but evolves over the lifespan [10].

Despite enormous progress in neuroscience in the last few decades, so far, the exact mechanism of epileptogenesis is not known [11]. Among the events that occur in response to the initial insult, neuronal cell death has received significant attention as the propagating factor that may lead to the epileptic condition. However, its actual role in epileptogenesis is still controversial. Neuronal cell death can be presented either as a consequence of epileptic seizure only, or both, a cause and a consequence, as proposed by the increasing evidence [3, 9]. 

Seizures can result in neuronal cell death through dynamic processes that might include genetic factors, excitotoxicity induced mitochondrial dysfunction, altered cytokine levels, oxidative stress, and changes in plasticity or activation of some late cell death pathways [12-14]. It was suggested that oxidative damage and consequently neuronal cell death have common pathogenic processes that can contribute to epileptogenesis and to initiation and propagation of spontaneous recurrent epileptic seizures [15].

Initial brain insult causes a cascade of structural, physiological, and biochemical changes, such as altered cerebral blood ﬂow and vasoregulation, disruption of the blood brain barrier, increased intracranial pressure, focal or diffuse ischemic haemorrhage, inﬂammation, necrosis and disruption of ﬁber tracts and blood vessels [16]. In posttraumatic epilepsy, for example, it was suggested that after intracranial haemorrhage, red blood cells break down and release haemoglobin (Hb) and iron ions. Consequently ROS are generated by iron- or Hb-mediated reactions [16, 17]. Increased production of ROS induces a cascade of cellular and molecular changes that might lead to hyperexcitability of neurons or to neuronal death [18]. The hypothesis of neuronal death caused or propagated epilepsy is further supported by the fact that surgical removal of a damaged hippocampus improves the condition of epilepsy patients [19]. Therefore nowadays, accumulating evidence suggests that neuronal cell death may be both a cause and a consequence of epileptic seizures [3].

### The Phase of Spontaneous Recurrent Seizures - Chronic Epilepsy

The major mystery that still has to be explained is how the initial injury can produce these long-term changes in neuronal excitability that lead to spontaneous recurrent seizures of acquired epilepsy. 

As discussed above, brain insult results in increased oxidation of cellular macromolecules prior to the death of vulnerable neurons. At this stage endogenous antioxidant defence system prevents seizure-induced neuronal death [20-22]. Oxidative stress has been suggested to be a significant consequence of excitotoxicity that plays a critical role in epileptic brain damage. Excessive ROS production leads to increased intracellular concentration of Ca^2+^ ions through mechanisms discussed in the following section. Ca^2+^ overload is involved in seizure-induced neuronal death, as well as in necrosis and apoptosis [23].

It is suggested, that during the injury phase of seizures, Ca^2+^ concentration may reach high levels. If concentrations are moderately elevated, neurons are subjected to long lasting neuroplasticity changes. However, in case of very high Ca^2+^ concentrations, cell death is induced. Ca^2+^ concentration remains elevated during the latent phase. Therefore, many second messenger effects, which can produce long-lasting plasticity changes in these neurons, are initiated [7]. High Ca^2+^ concentration in the epileptic neurons remains elevated also during the chronic epilepsy phase, and plays a role in maintenance of the spontaneous recurrent seizures. It can alter GABA_A_ receptor recycling which can serve as a possible mechanism for the effect of Ca^2+^ on altering neuronal excitability [24]. Furthermore, increased intracellular Ca^2+ ^concentration can effect numerous cell physiological processes, including gene transcription, protein expression and turnover, neurogenesis, neuronal sprouting, and many others [25]. 

## SIGNALLING ROLES OF Ca^2+^ AND ROS

ROS play an important role in cell signalling [26]. Mitochondria are important players in maintaining cell Ca^2+^ homeostasis and serve as Ca^2+^ buffer. Physiological increases in mitochondrial Ca^2+^ lead to the activation of tricarboxylic acid (TCA) cycle enzymes including isocitrate dehydrogenase, α-ketoglutarate dehydrogenase, maleate dehydrogenase, as well as succinate dehydrogenase [27]. Their activation results in increased concentrations of reduced substrates for oxidative phosphorylation (NADH and FADH_2_). Therefore, physiological increase in mitochondrial Ca^2+^ results in enhanced respiratory chain activity, increased proton pumping and consequently generation of ROS. The reciprocal interaction between Ca^2+^ modulated ROS production and ROS modulated Ca^2+^ signalling underlies the concept of ROS and Ca^2+^ crosstalk. Ca^2+^ can alter ROS production by simply increasing metabolic rate *via *TCA cycle stimulation [28]. Moreover, Ca^2+^ can activate nitric oxide synthase (NOS) and generate nitric oxide. Nitric oxide has been shown to inhibit complex IV, which can in turn lead to ROS production at the Q_0_ site of complex III [29]. Nitric oxide has also been shown to elicit synaptic glutamate release and therefore contribute to further excitotoxicity in neighbouring cells [29]. Hence, Ca^2+^ may stimulate oxidative phosphorylation electron flux on one side, and partial inhibition of the electron transport chain on the other. Inhibition of electron transport chain could lead to an increased possibility of electron slippage to oxygen. 

Just as Ca^2+^ plays a role in the production of ROS, cellular redox state can significantly modulate Ca^2+^ signalling. Moreover, protein redox state modulates many cellular processes. Changes in structure caused by oxidizing amino acid residues can change the activity of enzymes and ion transporters [30]. In the context of Ca^2+^ signalling, redox state and ROS can stimulate, as well as inhibit Ca^2+^ channels/pumps/exchangers, generally increasing Ca^2+^ channel activity and inhibiting Ca^2+^ pumps [31]. Precisely, ROS can directly, or indirectly through changes in cell redox homeostasis, oxidize redox-sensing thiol groups on the ryanodine receptors leading to channel stimulation. Ryanodine receptors are the primary Ca^2+^ release channel involved in the sarcoplasmic Ca^2+^ release. Furthermore, ROS derived stimulation of inositol-1,4,5-triphosphate receptor channels and inhibition of sarco/endoplasmic reticulum ATPase and plasma membrane Ca^2+^ ATPase, results in increased mitochondrial Ca^2+ ^concentrations. Inositol-1,4,5-triphosphate receptor channels are the primary Ca^2+ ^endoplasmic reticulum release channels, while sarco/endoplasmic reticulum and plasma membrane Ca^2+^ ATPase are Ca^2+^ re-uptake pumps. Furthermore, there is also an important plasma membrane Na^+^/Ca^2+^ exchanger [31]. Its function is also modulated by oxidation. Currently, its exact role is still unknown, as ROS can both, stimulate and decrease its activity.

The stimulation of mitochondrial Ca^2+^ signals by ROS and increased ROS generation resulting from increased mitochondrial Ca^2+^ can lead to a positive feedback loop. So Ca^2+^ can pose both, a physiological and a pathological effect of mitochondrial function.

### Oxidative Stress as a Disturbance of Physiological Signalling Roles of Ca^2+^ and Free Radicals

Oxidative stress is defined as an imbalance between the production of ROS on one side and endogenous antioxidant and repair capacity on the other, in favour of the former [32].

As Ca^2+^ buffer, mitochondria can suffer Ca^2+^ overload, which may lead to free radical production. It is thought, that a delicate balance exists between moderate ROS production to modulate physiological signalling, and overproduction of ROS resulting from mitochondrial Ca^2+^ overload that ultimately leads to oxidative stress, cellular damage, and eventually cell death. Ca^2+^overload triggers the opening of the mitochondrial permeability transition (MPT) pores which is linked to apoptosis *via *the mitochondrial pathway or necrosis due to mitochondrial damage [33]. This confirms the complex interdependence between mitochondrial energy production, Ca^2+^ uptake, ROS generation, ROS neutralisation, and redox signalling. While physiological increases in mitochondrial Ca^2+^ are beneficial for metabolism, overload is detrimental to mitochondrial function and can become pathological. 

### Possible Role of Free Radical Homeostasis Disturbance in Epileptogenesis

The role of free radical homeostasis in neuronal disorders is of major interest, since cells in the CNS are particularly vulnerable to the harmful effects of ROS and reactive nitrogen species (RNS). In addition, antioxidant defence mechanisms in CNS are particularly poor. This is especially important because the brain is rich in mitochondria and is characterised by high aerobic metabolic activity, high oxygen consumption, high ratio of membrane surface area to cytoplasmic volume, high concentration of polyunsaturated fatty acids, and a neuronal network vulnerable to disruption [34]. The brain is also rich in iron, and brain damage releases iron ions capable of catalyzing free radical reactions [35]. Furthermore, superoxide radicals can arise also from the auto-oxidation of catecholamines and in the cytoplasm by enzymes, such as xanthine oxidase [36]. It is important to note that endogenous antioxidants and repair capacity weaken with aging [32]. Among brain cells, neurons are particularly vulnerable to oxidative insults due to low levels of antioxidant enzymes, especially catalase (CAT) and glutathione peroxidase (GPX), and of nonenzymatic antioxidants, namely vitamin E and glutathione (GSH) [37]. 

In the CNS there are two major contrary acting neurotransmitters: the excitatory acting glutamate and the inhibitory acting γ-amino butyric acid (GABA). The excitatory glutamate, which could act toxically at higher concentrations, is thought to be one of the major contributors in oxidative stress development [38]. 

Oxidative stress is capable of damaging diverse cellular components or molecular targets, including nucleic acids, lipids, proteins, and carbohydrates [23]. Oxidation of monosaccharide sugars results in the formation of oxaldehydes, which can contribute to protein aggregation [39]. 

Free radicals may trigger disruption of nucleinic acids. They can break DNA strands or directly modify purine and pyridine bases, which leads to deletions and other mutations. DNA damage activates the DNA repair enzyme poly-ADP-ribose polymerase-1 (PARP-1), which overactivation depletes its substrate, nicotinamide adenine dinucleotide (NADH), slowing the rate of glycolysis, electron transport, and ATP formation, eventually leading to functional impairment or cell death [40]. There is also mtDNA, which is even more vulnerable target for free radical damage because of diminished repair mechanisms and lack of histones and due to its vicinity to the site of ROS generation. mtDNA disruption may lead to mitochondrial dysfunction resulting in disturbed cell function. Finally, RNA is the most susceptible to oxidative damage, since it is single stranded, not protected by hydrogen bonding, and less protected by proteins. RNA damages may result in altered proteins or dysregulation of gene expression [41]. One of mitochondrial disorders is the myoclonus epilepsy with ragged red fibers (MERRF) syndrome that is linked to point mutations in the mitochondrial tRNALys gene [42, 43]. Partial seizures are also frequently noticed in mitochondrial encephalopathy with lactic acidosis and stroke-like episodes (MELAS) syndrome, which is associated with mutations in the mitochondrial tRNALeu gene [44, 45].

Polyunsaturated fatty acids in lipoproteins and phospholipids of biological membranes are also highly susceptible to oxidative damage, which leads to lipid peroxidation (LP). LP disrupts biological membranes and is thereby highly deleterious to their structure and function [46]. A large number of by-products are formed during this process, since unsaturated hydroperoxides generated by peroxidation of polyunsaturated fatty acids can break down to form different reactive aldehydes; the most known is malondialdehyde (MDA). Reactive aldehydes can bind covalently to proteins, thereby altering their function and inducing cellular damage [3]. 

Of particular importance is also protein redox state, as already described above in the section describing signalling roles of Ca^2+^ and ROS. Another possible consequence of increased ROS production is impairment of the Na^+^/K^+^-ATPase activity, which normally maintains ionic gradients of neuronal membranes. These chemical and electrical gradients generate the background for electrical activity, which is essential for normal nervous system functions. A decrease in Na^+^/K^+^-ATPase activity might decrease the convulsive threshold and therefore additionally lead to an increase in the release of excitatory neurotransmitters, such as glutamate and aspartate, or to a decrease of inhibitory neurotransmitters, such as GABA.

Glutamate is mainly present in the intracellular space. Increased glutamate concentration in the extracellular compartment can be toxic to neurons [47]. Increase in the extracellular concentration of glutamate in the case of CNS injury or disease has been linked to a number of potential mechanisms including excessive release and impaired cellular uptake. ROS generation can also support the induction of seizure activity by direct inactivation of glutamine synthetase (GS), thus permitting an excessive increase in glutamate. Excessive glutamate receptor activation caused either by glutamate or glutamate receptor agonists can induce neurotoxicity, which is described by the term excitotoxicity. This is manifested in excessive stimulation of glutamate receptors, namely NMDA (N-methyl-D-aspartate) and AMPA (α-amino-3-hydroxy-5-methyl-4-isoxazolepropionic acid) receptors, and an associated overwhelming increase in free cytosolic and mitochondrial Ca^2+^ concentration. 

Sometimes Ca^2+ ^can reach high levels in neuronal cells already during the injury phase. It is anticipated that prolonged seizures, like status epilepticus, result in sufficient production of ROS to overwhelm the endogenous mitochondrial antioxidant defences [23].

**Fig. (1):**
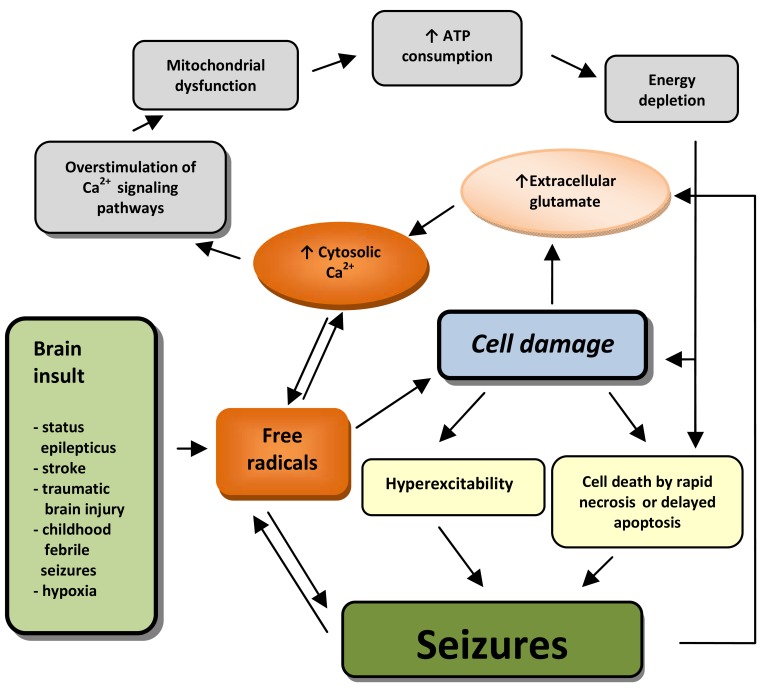
A schematic presentation of proposed mechanism of cell damage and seizure generation in acquired epilepsy. Seizures or initial brain insult lead to accumulation of free radicals. Seizures alone, or through cell damage, cause hyperexcitability and increased extracellular glutamate concentration, which results through increased cytosolic Ca^2+^ concentration and consequently overstimulated Ca^2+^ signalling pathways in mitochondrial dysfunction, increased ATP consumption and energy depletion. This is associated with cell damage and necrotic or delayed apoptotic cell death. Cell death in itself is again considered as a cause of seizures. Beyond that, hyperexcitability may lead to lower seizure threshold, which can result in propagation reaction.

More usually, during the injury phase, Ca^2+^ levels are insufficient to cause cell death. However, later during the latent phase, Ca^2+^ remains elevated and initiates many effects mediated by second messengers and consequently causes long lasting changes in neurons, including their death (see Fig. **1**). If neuronal cells survive latent phase, Ca^2+^ still remains elevated during chronic phase and therefore plays unique role in maintaining spontaneous recurrent seizures [7].

Overall, all oxidative modiﬁcations may disturb the function of enzymes, receptors, neurotransmitters, and structural proteins, and therefore could contribute to progressive cell decline, aberrant neuroplasticity changes, and ultimately even cell death [23].

### Mitochondrial Involvement

**Fig. (2):**
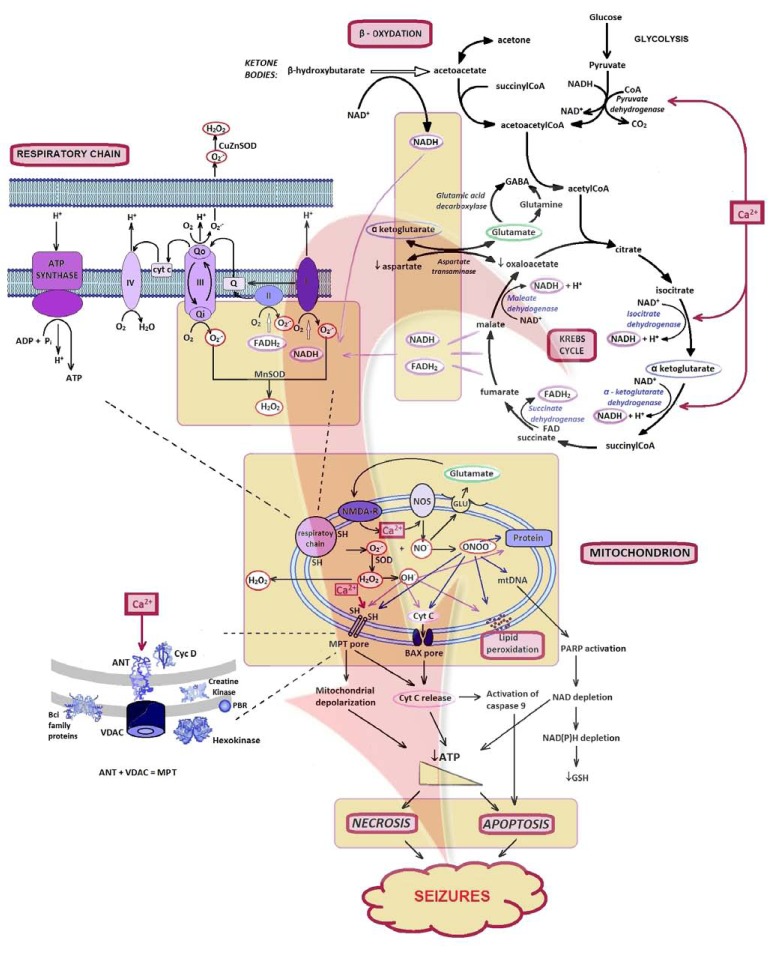
Assumed role of mitochondria, Ca^2+^, and ROS in neuronal excitotoxicity and epileptogenesis. After increase of mitochondrial Ca^2+^ inﬂux, mediated by various mechanisms, the generation of both ROS and RNS elicit downstream cell death signalling. The Ca^2+^ passage can lead to propagation and ampliﬁcation of mitochondrial Ca^2+^ overload. The mitochondrial respiratory chain is shown with complexes I, II, III, IV, and V. ROS and RNS can react with thiol groups (-SH) and subsequently cause opening of the MTP pore and Bax pore. One of the consequences is release of cyt-c across the outer membrane, which is the major initiation step in a cascade reaction of cell apoptosis. The cell death pathway may switch between apoptosis and necrosis, in relation to the intracellular ATP availability. Symbols legend: Ac-CoA: Acetyl coenzyme A; ANT: Adenine nucleotide translocase; Cyt-c: Cytochrome c; FADH_2_: Flavin adenine dinucleotide phosphate; GSH: Reduced glutathione; I–V: complexes I–V; MnSOD: Manganese superoxide dismutase; MPT: mitochondrial permeability transition; mtDNA: Mitochondrial deoxyribonucleinic acid; NAD: Nicotine adenine dinucleotide; NO: Nitric oxide; NOS: NO synthase; PARP: Poly-ADP-ribose polymerase; PBR: Peripheral benzodiazepine receptor; RNS: Reactive nitrogen species; ROS: Reactive oxygen species; VDAC: Voltage-dependent anion channel.

The main mitochondrial function is the production of cellular energy in the form of ATP by mitochondrial respiratory chain through oxidative phosphorylation by five multienzyme complexes located in the mitochondrial inner membrane [29]. Complexes I-IV are oxidoreductases which participate in the transfer of electrons from reduced substrates to oxygen and create an electrochemical proton gradient across the mitochondrial inner membrane. Complex V (ATP synthesis) couples proton reuptake with adenosine diphosphate (ADP) phosphorylation in the matrix to generate ATP [48] (Fig. **2**). In addition to the energy production, mitochondria also play a crucial role in the maintenance of intracellular Ca^2+^ homeostasis, neurotransmitter biosynthesis, generation of ROS and mechanisms of cell death [48]. 

More recently, accumulating evidence suggests that mitochondria are also involved in acquired forms of epilepsy. Increase in mitochondrial oxidative stress and subsequent damage to cellular macromolecules have been demonstrated following repeated or prolonged seizures like status epilepticus and other injuries, such as hypoxic-ischemic insults, traumatic brain injury, or viral infection [23]. Impairment of mitochondrial function and increased ROS production has recently been demonstrated in human [49-51] and in several animal models of epileptic seizures [23, 52-54].

These changes strongly affect neuronal excitability and synaptic transmission, which is proposed to be highly relevant for seizure generation [3, 49]. Mitochondria could be involved in pathways leading to neuronal cell death, characteristic for the areas of epileptogenesis. Status epilepticus induced in experimental models by kainic acid or pilocarpine, are known to activate programmed cell death mechanisms [55]. Moreover, increased production of ROS is a feature of partially respiratory chain-inhibited mitochondria [56]. 

Mitochondrial proteins can be modified by carbonylation, nitration, S-glutathionylation, or S-nitrosylation. Function of many metabolic enzymes in the mitochondrial extracellular compartment, including ATPase, cytochrome c oxidase, NADH dehydrogenase, and NADH oxidase, can be consequently altered [57]. Oxidation of adenine nucleotide translocator (ANT) impairs the influx of adenosine diphosphate (ADP) into the matrix for ATP synthesis. Oxidation of manganese superoxide dismutase (MnSOD) can further compromise antioxidant capacity and lead to further oxidative stress [58]. Increased production of ROS directly down-regulates proteins of tight junctions and activates matrix metalloproteinases that contribute in the opening of the blood brain barrier [59]. This allows the entry of neurotoxins and inﬂammatory cells, which potentiates already existing oxidative stress. 

Oxidative stress emerges as a common underlying cause of blood brain barrier dysfunction [60]. If disrupted, transport of molecules between blood and brain is altered in both directions. Increased oxidative mtDNA damage, mitochondrial hydrogen peroxide production, and alterations in the mitochondrial base excision repair pathway have been noted in the rat hippocampus after a period of three months following status epilepticus [15]. One of the consequences of oxidative damage is the release of mitochondrial cytochrome c into the cytosol, most probably by MPT pore. The MPT pore is an assembly of pre-existing proteins of the inner and outer mitochondrial membrane formed as a high conductance channel. It allows Ca^2+^ to be released from the mitochondria [61, 62]. MPT pore is formed by the apposition of the voltage-dependent anion channel (VDAC) in the outer membrane, the ANT in the inner membrane and the soluble matrix protein cyclophilin D [62]. The MPT pore is triggered by high Ca^2+^ concentration and other stimuli, including ROS, which may promote MPT pore by causing oxidation of thiol groups on the ANT. Likewise, ADP, reduced GSH, pyridine nucleotide pool, and acidic pH are all inhibitors of MPT opening [31]. 

Overall, as all other processes, also MPT triggering by ROS is potentiated by increased Ca^2+^. Ca^2+ ^is believed to be the coordinating signal to cytochrome c release. Bax, a proapoptotic member of the Bcl-2 family, interacts with MPT pore to induce permeability transition and cytochrome c release which is a key event in apoptosis [63]. Besides cytochrome c, opening of the MPT also leads to mitochondrial depolarization and loss of matrix solutes including GSH, ADP/ATP, etc. Cytochrome c released into the cytoplasm, further triggers the complex intrinsic mitochondrial pathway of apoptosis as shown in Fig. (**2**). The cell death pathway may switch between apoptosis and necrosis, depending on the availability of intracellular ATP. Whereas apoptosis is the principal cell death pathway in the presence of sufficient ATP, overwhelming depletion of ATP results in necrotic cell death. In general, mitochondrial Ca^2+^ overload and consequent mitochondrial dysfunction appear to be combining factors in excitotoxicity. They are playing key roles in cell death [64]. 

Epileptic seizures can occur as a presenting sign of mitochondrial dysfunction in the central nervous system. This is seen in the occurrence of epileptic seizures in mitochondrial diseases arising from mutations in mtDNA or nuclear DNA [49]. In addition, systemic administration of mitochondrial toxins, such as 3-nitropropionic acid and cyanide, inhibits the functions of the mitochondrial respiratory chain that can compromise cellular energy metabolism and induce seizures in animal models [65, 66]. These accumulating evidences implicate that both, mtDNA mutations and exogenous mitochondrial toxins cause mitochondrial respiratory chain dysfunction which is associated with at least some of the mechanisms of epileptogenesis. Recently, evidence for a more general involvement of mitochondria, also in sporadic forms of epilepsy has been provided [67, 68]. Collectively, experimental and human epilepsy studies prove that mitochondria are intimately involved in pathways leading to neuronal cell death [49]. Therefore, increased mitochondrial oxidative stress and mitochondrial dysfunction may be the final common pathway that underlines all these neuropathological conditions and contributes to epileptogenesis [69]. In this way it could be capable of leading to chronic acquired epilepsies.

Mitochondrial dysfunction has gained considerable interest as a potential source of ROS and consequently a cause of epileptic seizures, especially in therapy-resistant forms of severe epilepsy.

Recent proteomic studies confirmed that certain mitochondrial components are altered following seizure activity and that brain injury may be explained by these alterations [70]. Hence, mitochondria can be considered as a target for potential neuroprotective strategies in epilepsy. 

## ANTIEPILEPTIC DRUG THERAPY AND OXIDATIVE STRESS

Antiepileptic drugs (AEDs), at least in part, impair antioxidant systems. The ability of antioxidants to attenuate seizure generation and the accompanying changes in oxidative burden, further support an important role of antioxidants as having putative antiepileptic potential. Epilepsy is usually well controlled by presently available drugs. However, in 20 to 30% of patients seizures are not controlled with available medication. The majority of these patients suffer from focal form of epilepsy. The areas of epileptogenesis in these cases are usually characterised by cell loss. One of the most frequent and devastating forms of epilepsy involves the development of an epileptic focus in temporal lobe structures. While the granular cell layer is relatively preserved, the progressive loss of pyramidal cells of the CA1, CA3 and CA4 layers are the neuropathological hallmarks. Progressive cell loss is suggested to be a major reason why seizures of temporal lobe origin become particularly resistant to antiepileptic drug therapy at later stages of the disease [71].

Since antiepileptic treatment, to a various extent impairs the intrinsic ability to counteract oxidative stress by reducing endogenous antioxidants or increasing free radical production, progression of epilepsy was investigated in various clinical studies. 

### Influence of AEDs on Antioxidants and Markers of Oxidative Stress in Patients with Epilepsy

The majority of oxidative stress measurements in patients with epilepsy have been performed on peripheral tissues, such as plasma, serum or red blood cells. However, oxidative stress may originate from various sources in the body and peripheral measurements might not necessarily accurately reflect the oxidative stress in the CNS. Nevertheless, studies on rat models of FeCl_3_/kainate brain injection induced seizures indicate that lipid peroxidation products were increased not only in the CNS, but also in blood [72, 73]. It should be mentioned that also the reverse situation is possible. In a study by Aytan *et al*., it has been established that peripheral oxidative stress can change oxidative status of CNS. In a rabbit model, feeding with high cholesterol diet caused an increase in serum MDA, which clearly correlated with an increase in protein oxidation parameters in the brain [74].

Increased activities of SOD [75, 76] and CAT [75, 77], and decreased activities of GPX [75, 78] and glutathione reductase (GR) [79] was observed in numerous studies in drug-naive patients with epilepsy. In a recent study, LP and percentage haemolysis in patients with epilepsy was higher compared to controls [79]. Furthermore, erythrocyte GR and plasma ascorbate and vitamin A concentrations were lower [79]. In the majority of these studies also increased markers of lipid peroxidation were noted [75, 77-82].

On the other hand, there are also studies, with very weak (unchanged SOD, CAT, GPX and GR activities) [78, 79, 82-84] or even opposite (decreased lipid peroxidation markers) [85] association of oxidative stress with epilepsy.

AEDs have diverse effects on the antioxidative system [86-88]. Some of them, especially from the older generation of AEDs (such as CBZ and VPA) may trigger oxygen-dependent tissue injury by several mechanisms [89]. Oxidative injury may play a role in the initiation and progression of epilepsy, therefore therapies aimed at reducing oxidative stress may ameliorate tissue damage and favourably alter the clinical course. Peroxidation of membrane lipids caused by an increase in generation of free radicals or decrease in the activities of antioxidant defence systems has been suggested to be critically involved in the seizure control.

Oxidative stress in patients with epilepsy treated with AEDs was assessed by measuring serum, plasma, erythrocyte, leukocyte or urine markers of macromolecular damage (MDA, RCD, P-SH, 8-OHdG, 15F-2t-isoP) and antioxidative enzyme activity (SOD, GPX, CAT, GR) (Table **1**). 

The role of valproic acid (VPA) in exacerbation of oxidative stress is supported by reduced total antioxidant capacity (TAC) [86, 90] and enhanced total oxidative status (TOS) [90], reported for patients treated with VPA. Erythrocyte GPX [91, 92], GR [93] and serum Se [94], uric acid and albumin [80], which are considered endogenous enzymatic and non-enzymatic antioxidant molecules, were found to be reduced in children and adults treated with VPA. Furthermore, in studies in patients with epilepsy, treated with VPA, increased levels of oxidative macromolecular damage markers, such as MDA [76, 86, 90, 91] and 15F-2t-isoP [95], which are markers of enhanced lipid peroxidation and increased levels of 8-OHdG [90, 96], which is a marker of nucleic acid damage, were observed. Similarly, enhanced lipid peroxidation [80] and decreased SOD and GPX [92] was seen in studies with phenobarbital.

Elevated levels of lipid hydroperoxide were observed in phenytoin treated patients with epilepsy [88, 97]. Treatment with phenytoin was also associated with lower TAC and GSH. The role of carbamazepine (CBZ) in generating free radicals and consequently causing neuronal damage is not so evident. CBZ enhanced plasma LP in adolescent and adult patients [86] and caused marked increase in serum total peroxide levels in children [80]. Lower TAC [86] and GSH [98] in plasma, and GPX and CAT in erythrocytes were also observed [92]. In accordance with its pro-oxidative action, decreased SOD [92] and increased 8-OHdG [96] and nitrite/nitrate in erythrocytes were also observed. However, there are also studies which do not confirm CBZ pro-oxidative action [83, 86, 91, 95] and even studies with anti-oxidative action [88, 93, 99]. 

Long-term use of certain AEDs has been proposed to increase free radical formation and cause oxidative damage in neurons [90, 99, 110]. As demonstrated in Table **1**, this is particularly true for older AED generation, namely VPA, phenytoin and CBZ. Exacerbation of oxidative stress during treatment with AEDs could also be one of the reasons for pharmacotherapy resistant epilepsy. These pro-oxidative effects might lead to enhancement of seizure activity through increased hyperexcitability and/or the induction of neuronal damage, which can result in loss of AEDs efficacy or apparent functional tolerance and undesired side effects. Such functional tolerance may lead to complete loss of AED activity and cross-tolerance to other AEDs Tolerance develops to some AED effects much more rapidly than to others. It may lead to attenuation of side effects and also to loss of efficacy and is reversible after discontinuation of drug treatment [111]. Experimental evidence indicates that almost all first-, second-, and third-generation AEDs lose their antiepileptic activity during prolonged treatment, although to a different extent [111].

Many conventional anticonvulsants, namely phenobarbital, CBZ, and VPA are metabolized to generate reactive metabolites with capability of covalent binding to macromolecules [86, 112]. Therefore, the AEDs may not only suppress the epileptic seizures but also elicit systemic toxicity. There are at least two potential mechanisms by which the free radicals produced by oxidation of phenolic metabolites could lead to toxicity, by generating ROS and/or by covalent binding of their metabolic intermediates to proteins or other vital macromolecules [89, 112, 113]. 

Unfortunately, till now little is known about pro-oxidative or neuroprotective effects of newer second and third generation AEDs. Oxcarbazepine (OXC) and levetiracetam (LEV) are classified as newer, second generation AEDs [106]. As seen in the Table **1**, OXC antiepileptic actions could be due to its neuroprotective effects. Arhan *et al*. have shown that OXC decreases serum lipid peroxidation and nitrite/nitrate concentrations [101]. On the other hand, Ozden *et al*. found LEV to enhance lipid peroxidation [108]. To evaluate possible neuroprotective effects of newer AEDs more investigations need to be done in clinical practice on patients or on animal models.

### Effects of AEDs on Oxidative Stress Markers in Different Animal Models of Seizures and Epilepsy

The generation of seizures is associated with changes in the intracellular levels of antioxidants and oxidants. Various experimental animal seizures models have been designed and investigated specifically for the evaluation of the role of various AEDs on modulating oxidative stress markers. The sites of seizure generation are not uniform in various models. 

In numerous studies generation of free radicals was increased following pentylenetetrazol (PTZ) kindling, due to increased cytosolic Ca^2+^ concentrations. PTZ is best known for its use in screening antiepileptic drugs [104]. It is a tetrazole derivate with convulsant actions in mice, rats, cats, and primates, presumably by impairing GABA-mediated inhibition by action at GABA receptor. PTZ is considered a GABA antagonist. 

Blood and brain toxicity after PTZ induced seizures and kindling were investigated in a rat model [125]. LP levels of plasma, erythrocyte, and brain cortex and brain microsomal fraction were increased by PTZ administration. Additionally, increased serum NO levels and accumulation of hydroxyl radicals were reported, while plasma and brain enzymatic and nonenzymatic antioxidants (GPX, vitamin E) were decreased. 

Another often used model is pilocarpin induced model of seizures and epilepsy. Pilocarpine is a cholinergic agonist commonly used to induce seizures and an epilepsy-like status in rodents. The pilocarpin model of epilepsy relates both, to a model of acute induced seizures culminating with a prolonged episode of status epilepticus, as well as to a model of chronic spontaneous seizures [126]. During status epilepticus induced by pilocarpine in combination with lithium, hyper metabolism occurs with increased glucose consumption, which results in abnormal respiratory chain and neuronal damage [127]. As reported by Bellissimo *et al*., in pilocarpine model of epilepsy, in rats presenting status epilepticus or spontaneous seizures, decreased activity of SOD and increased levels of hydroperoxides in the hippocampus were observed [128]. To follow the evolution of neuronal damage caused by oxidative stress, cellular activation (investigated by fos protein expression and glucose utilization) and stress response (investigated by heat-shock protein (HSP72), immunoreactivity, and acid fuchsine staining) in lithium-pilocarpine seizure adult rat models of various duration, were analysed [129]. These investigations proved that regions with the largest metabolic activation coincided with the highest stress response and were consequently most heavily damaged [129].

In animal models of seizures and epilepsy, various AEDs were investigated. For instance, topiramate (TPM), is a voltage-gated Ca^2+^ channel inhibitor. The effects of TPM administration on pentylenetetrazol-induced rat model of seizures showed that lipid peroxidation levels in serum and kidneys and the nitric oxide levels in kidneys were decreased by TPM, while SOD and CAT activities in the kidneys were increased. GPX activity was not affected by pentylenetetrazol or TPM. It was concluded that TPM has protective effects on pentylenetetrazol-induced toxicity by inhibition of free radicals and by support of the antioxidant redox system [117, 119]. Many antiepileptic drugs have been investigated for their ability to attenuate the oxidative stress in animal models of seizures, the details of which are given in Table **2**.

### Effects of AEDs on Markers of Oxidative Stress Studied *in vitro* in Primary Rat Astrocytes

Astrocytes together with microglia constitute more than 90% of the total cell population in the adult brain. They are situated in key pivotal places in the CNS that might play an important role in epilepsy in several ways. They support neurons by providing different trophic factors [130]. It has been found that their function is not just to provide support to neurons, but also to play several other very important roles [131]. Astrocytes express receptors for different neurotransmitters [132]. For instance, they can respond to a local application of glutamate with a calcium elevation that travels as a wave [133]. These data suggest that astrocytes signal using spike-like calcium transients, which can travel over long distances and serve as a tool for neuron-astrocyte communication. 

During normal brain function activity, astrocytes play a major role in the clearance of glutamate that is released from the nerve terminal into the extracellular space. Hence, when this astrocytes function is damaged, it may result in oxidative damage and associated epileptogenesis propagation or even its initiation. Therefore, studies on astrocytes might serve as a useful cell model to study the effects of AEDs on redox homeostasis.

Till now, only results from *in vitro* studies on primary rat astrocyte cell cultures are published [130, 134, 135]. Astrocytes have higher concentration of reduced glutathione and antioxidant enzymes. They pose glutamine synthetase, which is very sensitive to oxidative stress [35, 134]. On the other hand, it has been observed that in some pathological conditions astrocytes may contribute to neurological damage by increasing the production of ROS [35].

CBZ, OXC and TPM were demonstrated to initiate an oxidative process in primary cultures of rat cortical astrocytes. Both ROS and NO were shown to be involved (Table **3**). These results indicate that the newer, second generation AEDs change the examined metabolic activities to a much lesser extent, at least at therapeutic concentrations. Newer, second generation AEDs express neuroprotective effects on glial cells and, when used at an appropriate cell-specific concentrations, may be well tolerated by cortical astrocytes. Especially at higher AED concentrations, gabapentin (GBP), lamotrigine (LTG), tiagabine (TGB), and LEV seem to be better tolerated than are CBZ, TPM, and OXC. GBP, LTG, TGB, and LEV toxic effects on astrocytes were observed only at higher concentrations. 

According to these findings, LEV and TGB may be considered as effective promising drugs in the treatment of epilepsy. However, a few other studies also claim a neuroprotective role for some older AEDs through antioxidative pathways. VPA for instance, was shown to be protective against oxidative stress in both, *in vitro* and *in vivo* models of epilepsy. It was suggested that VPA increases levels of glutathione [136, 137]. Thus, AEDs have been shown to contribute to both pro- and anti-oxidant activities. Their role in exacerbation of oxidative stress is still to be investigated. 

## CONCLUSION

Since it has been shown that mitochondria are closely involved in pathways leading to neuronal cell death it seems reasonable to assume that mitochondrial dysfunction has a considerable role in epileptogenesis. 

Additionally, ROS have an important role in cell signalling. Mitochondria are crucial players in maintaining cell Ca^2+^ homeostasis and serve as Ca^2+^ buffer. As a consequence of Ca^2+ ^influenced ATP production in the mitochondria, ROS are generated. It is proposed that Ca^2+ ^as well as ROS under physiological conditions work as a positive effectors and cellular signalling molecules, while their overload can lead to mitochondrial dysfunction, oxidative stress and cell damage. 

Oxidative stress is considered one of the mechanisms that could independently contribute to the disease progression, in addition to serving as processes that underlie neuronal injury. It could also cause mitochondrial dysfunction, cell damage and consequently epilepsy, since neuronal dysfunction or death could serve as the propagating factor that may lead to the epileptic condition.

Various AEDs have diverse effects on the antioxidative system and some of them, especially from the older generation may trigger oxygen-dependent tissue injury by several mechanisms. Their influence on oxidative status in patients with epilepsy was extensively reviewed. Long-term use of certain AEDs has been proposed to increase free radical formation and cause oxidative damage in neurons. Furthermore AEDs pro-oxidative effects might lead to enhancement of seizure activity, which can result in loss of AEDs efficacy or apparent functional tolerance, pharmacotherapy resistance and undesired side effects. Convincing experimental evidence indicates that almost all first-, second-, and third-generation AEDs lose their antiepileptic activity during prolonged treatment, although to a different extent. Some reactive AED metabolites are capable of covalent binding to macromolecules and therefore not only suppress the epileptic seizures but also elicit systemic toxicity.

Since neuronal loss seems to be one of the major neurobiological abnormalities in the epileptogenic and epileptic brain, neuroprotective treatment during the epileptic process should result in milder structural damages, reduced epileptogenesis and less severe cognitive decline. 

The latent period during the epileptogenesis, including the concept of ROS and Ca^2+^ crosstalk represents one of the more recent, still largely untapped potential, where targeted therapies could act to inhibit epileptogenesis, thereby preventing the development of acquired epilepsy. Recent studies have confirmed elevated Ca^2+^ during the injury phase. It remains significantly elevated in epileptogenesis and is chronically elevated in the chronic epilepsy phases of acquired epilepsy. 

Nowadays, rapid and more precise discovery of possible causes and better understanding of underlying mechanisms of epileptogenesis opens up countless new possibilities for epilepsy therapy. Future is currently reflected in the design of AEDs with anticonvulsant and at the same time also neuroprotective activity. One of the potential mechanisms can be modulation of ROS - Ca^2+^ crosstalk.

## Figures and Tables

**Table 1.** Effects of Antiepileptic Drugs on Antioxidants and Oxidative Stress Markers in Blood of Patients with Epilepsy

AED		Findings	Materials	Subject	References
**Valproic acid**	LP	↑	Erc	Childhood	[76, 99]
		↑	Plasma	Adolescent and adult	[86]
		↑/-/-	Serum	Childhood	[90, 91, 100]*/[101]
	*(15F-2t-isoP)*	↑	Urine	Childhood	[95]
	8-OHdG	↑	Serum	Childhood	[90]
		↑	Leukocytes	Adult	[96]
	TAC	↓	Serum	Childhood	[90]
		↓	Plasma	Adolescent and adult	[86]
	TOS/XO	↑/-	Serum	Childhood	[90]/[101]
	SOD	↓	Erc	Adult	[92]/[98]
		↑/-	Erc	Childhood	[89, 91]/[100]*
		-	Plasma	Childhood	[102]
	GPX	-/↓	Erc	Adult	[92]
		↓/↑/↓^a^	Erc	Childhood	[91, 99]/[93]*/[100]
		↑/-	Plasma/Serum	Childhood	[102]
		↑	Plasma	Adolescent and adult	[86]
	GR	↑^a^ /↓	Erc	Childhood	[100]/[93]
	Se	-/↓^a^	Plasma/Serum	Childhood	[99]/[94]
		-/↑	Serum	Adolescent and adult	[86, 92]
	Nitrite/nitrate	↑	Serum	Childhood	[103]
	NO	-	Serum	Childhood	[86]
	GSH	↓	Erc	Childhood	[100]*
		-	Plasma	Adult	[98]*
**Phenytoin**	LP	↑/↑	Serum	Adult/ Uncertain	[88]^/^[97]
	TAC	↓	Serum	Uncertain	[97]
	SOD	↑	Serum	Adult	[88]
	GSH	↓	Serum/Plasma	Adult	[88]/[103]*
**Carbamazepine**	LP	↑	Plasma	Adolescent and adult	[86]
	*(15F-2t-isoP)*	-	Urine	Childhood	[95]
	*(MDA)*	↓	Erc	Childhood	[93]
		-	Serum	Childhood	[104, 105]
	Total peroxide	↑	Plasma	Childhood	[80]
	8-OHdG	↑	Leukocytes	Adult	[96]
	TAC	↓	Plasma	Adolescent and adult	[86]
	SOD	-/↓	Erc	Adult	[92]
		↑/-	Erc	Childhood	[89]/[88, 103, 106]
**Carbamazepine**		↑	Serum	Adult	[88]
	GPX	-/↓	Erc	Adult	[92]
		↑/-	Erc	Childhood	[93, 106]*
		-	Plasma	Adolescent and adult	[86]
	CAT	↓	Erc	Adult	[92]
	Se	-	Erc/Plasma	Adolescent and adult	[86, 92]
	Nitrite/nitrate	↑	Serum	Children	[103]
	GSH	↓	Erc	Childhood	[100]*
		↓	Plasma	Adult	[98]*
**Phenobarbital**	Lipid hydroperoxide	↑	Plasma	Childhood	[80]
	SOD/GPX	↓	Erc	Adult	[92]
	GPX/GR	-/↑	Plasma	Childhood/Adult	[79, 107]/[35]
**Levetiracetam**	8-OHdG	-	Leukocytes	Adult	[96]
	LP *(15F-2t-isoP)*	↑	Urine	Adult	[108]
**Oxcarbazepine**	LP / Nitrite/nitrate	↓/↓	Serum	Childhood	[86]
	GPX/SOD	-/-	Erc	Adults	[109]

a Only in patients with a severe adverse effect related to valproic acid therapy

* Healthy control

↓ decreased; ↑ increased; - no significant changes observed; CAT: Catalase; Erc: Erythrocytes; GPX: Glutathione peroxidase; GR: Glutathione reductase; GSH: Glutathione; LP – Lipid peroxidation; MDA: Malondialdehyde; NO: Nitric oxide; SOD: Superoxide dismutase; TAC: Total antioxidant capacity; TOS: total oxidative status; 8-OHdG: 8-hydroxydeoxyguanosine; 15F-2t-isoP: 15-F(2t)-isoprostane.

**Table 2.** Effects of Antiepileptic Drugs on Oxidative Stress Markers in Various Animal Models of Seizures and Epilepsy

AED	Animals	Model	Marker	Findings	Materials	References
**Valproic acid**	Rats	Pilocarpine	CAT/NO	↓	Brain	[114]
			GSH	-		[114]
			Lipid peroxidation (MDA)	-		[114]
		PTZ-kindled	TAC/GSH/NO	↓/↑/-	Brain	[105]
**Carbamazepine**	Mice	PTZ-kindled	SOD/ CAT/GSH/ Lipid peroxidation (MDA)	-	Brain	[115]
**Trimetazidine**	Mice	PTZ-kindled	GSH	↑	Brain	[116]
			Lipid peroxidation (MDA)	↓		[116]
**Topiramate**	Rats	PTZ-induced	GPX	↑/-	Erythrocytes	[117]/ [118]
				-/↑	Brain microsomal	[117]
				-	Brain cortex	[81, 117, 119]
			GSH	↑↑	Erythrocytes	[117]
				↑	Brain cortex	[81, 117, 119]
				↑↑↑/↑↑	Brain microsomal	[81, 117, 119]
			Lipid peroxidation	↓?-/↓	Erythrocytes	[117]/ [118]
				↓↓	Brain cortex	[81, 117, 119]
				↓	Brain microsomal	[81, 117, 119]
	Mice	PTZ-kindled	SOD/CAT/GSH/Lipid peroxidation (MDA)	-/-/↓/↑	Brain	[120]
**Levetiracetam**	Mice	Pilocarpin	CAT/GSH/Lipid peroxidation (MDA)/Nitrite:Nitrate	↓/↑/↓/↓	Brain (hippocampus)	[121]
**Lamotrigine**	Mice	PTZ-kindled	SOD/CAT/GSH/Lipid peroxidation (MDA)	↑/↑/↑/↓	Brain	[115]
			SOD/CAT/GSH/Lipid peroxidation (MDA)	-/-/↓/↓	Brain	[120]
**Oxcarbazepine**	Mice	PTZ-kindled	SOD/CAT/GSH/Lipid peroxidation (MDA)	-/-/↓/↑	Brain	[120]
**Phenytoin**	Rats	Iron induced	Lipid peroxidation	-	Brain	[122]
**Zonisamide**	Rats	Iron induced	Lipid peroxidation (MDA)	↓	Brain cortex	[118, 123]
			8-OHdG	↓	Brain	[124]

↓ decreased; ↓↓ moderately decreased; ↑ increased; ↑↑ moderately increased; ↑↑↑ strongly increased; - no significant changes observed; ?- unclear influence; CAT: Catalase; GPX: Glutathione peroxidase; GR: Glutathione reductase; GSH: Glutathione; MDA: Malondialdehyde; NO: Nitric oxide; PTZ: Pentylenetetrazol; SOD: Superoxide dismutase; TAC: Total antioxidant capacity; 8-OHdG: 8-hydroxydeoxyguanosine.

**Table 3.** Effects of Therapeutic Concentrations of Antiepileptic Drugs on Primary Rat Astrocytes *in vitro*

AED	Ref. range (mg/L)	Conc. (mg/L)	C. viability (MTT)	C. toxicity (LDH)	GS	ROS	MDA	NO
**First-generation AEDs**								
**Carbamazepine**	4-12	10	↓↓	↑	↓	↑↑	↑↑	↑↑
**Second-generation AEDs**								
**Gabapentin**	2-20	10	-	-	-	-	↑	↑↑
**Lamotrigine**	2,5-15	10	-	-	-	↑	↑	↑↑
**Levetiracetam**	12-46	10	-	-	-	↑	-	
		50	↓	↑	↓	↑↑	↑	↑
**Oxcarbazepine**	3-3,5	1	↓	↑	↓	↑	↑	↑
		10	↓↓	↑↑	↓	↑↑	↑↑	↑↑
**Tiagabine**	5-20	10	-	-	-	↑	↑	-
**Topiramate**	5-20	10	↓	↑↑	↓	↑↑	↑↑	↑↑

Adapted from Pavone *et al*. [130]

↓ slightly decreased; ↓↓ moderately decreased; ↑ slightly increased; ↑↑ moderately increased (p < 0.01); - no significant changes observed (p > 0.01); LDH: Lactic dehydrogenase; GPX: Glutathione peroxidase; GS: Glutathione synthetase; MDA: Malondialdehyde; MTT: tetrazolium salt test; NO: Nitric oxide; ROS: Reactive oxygen species.

## References

[1] Delgado-Escueta AV (1999). Symptomatic lesional epilepsies. Jasper’s Basic Mechanisms of the Epilepsies.

[2] Stefan H, Halasz P, Gil-Nagel A, Shorvon S, Bauer G, Ben-Menachem E, Perucca E, Wieser HG, Steinlein O (2001). Recent advances in the diagnosis and treatment of epilepsy. Eur. J. Neurol.

[3] Patel M (2004). Mitochondrial dysfunction and oxidative stress: cause and consequence of epileptic seizures. Free Radic. Biol. Med.

[4] Scott RA, Lhatoo SD, Sander JW (2001). The treatment of epilepsy in developing countries: where do we go from here?. Bull. World Health Organ.

[5] Victor M, Ropper A (2001). Adams and Victor's principles of neurology.

[6] Bromfield EB, Cavazos JE, I JISJ (2006). Chapter 1. Basic Mechanisms Underlying Seizures and Epilepsy. An Introduction to Epilepsy.

[7] Delorenzo RJ, Sun DA, Deshpande LS (2005). Cellular mechanisms underlying acquired epilepsy: the calcium hypothesis of the induction and maintainance of epilepsy. Pharmacol. Ther.

[8] Pitkanen A, Sutula TP (2002). Is epilepsy a progressive disorder? Prospects for new therapeutic approaches in temporal-lobe epilepsy. Lancet Neurol.

[9] Pitkanen A, Lukasiuk K (2009). Molecular and cellular basis of epileptogenesis in symptomatic epilepsy. Epilepsy Behav.

[10] Scharfman HE (2007). The neurobiology of epilepsy. Curr. Neurol. Neurosci. Rep.

[11] Guerrini R, Casari G, Marini C (2003). The genetic and molecular basis of epilepsy. Trends Mol. Med.

[12] Ferriero DM (2005). Protecting neurons. Epilepsia.

[13] Haut SR, Veliskova J, Moshe SL (2004). Susceptibility of immature and adult brains to seizure effects. Lancet Neurol.

[14] Henshall DC, Simon RP (2005). Epilepsy and apoptosis pathways. J. Cereb. Blood Flow Metab.

[15] Chuang YC (2010). Mitochondrial dysfunction and oxidative stress in seizure-induced neuronal cell death. Acta Neurol Taiwan.

[16] Willmore LJ, Ueda Y (2009). Posttraumatic epilepsy: hemorrhage, free radicals and the molecular regulation of glutamate. Neurochem. Res.

[17] Rosen AD, Frumin NV (1979). Focal epileptogenesis after intracortical hemoglobin injection. Exp. Neurol.

[18] Gupta YK, Gupta M (2006). Post traumatic epilepsy: a review of scientific evidence. Indian J. Physiol. Pharmacol.

[19] Shin C, McNamara JO (1994). Mechanism of epilepsy. Annu. Rev. Med.

[20] Liang LP, Ho YS, Patel M (2000). Mitochondrial superoxide production in kainate-induced hippocampal damage. Neuroscience.

[21] Xavier SM, Barbosa CO, Barros DO, Silva RF, Oliveira AA, Freitas RM (2007). Vitamin C antioxidant effects in hippocampus of adult Wistar rats after seizures and status epilepticus induced by pilocarpine. Neurosci. Lett.

[22] Yamamoto HA, Mohanan PV (2003). Ganglioside GT1B and melatonin inhibit brain mitochondrial DNA damage and seizures induced by kainic acid in mice. Brain Res.

[23] Waldbaum S, Patel M (2010). Mitochondrial dysfunction and oxidative stress: a contributing link to acquired epilepsy?. J. Bioenerg. Biomembr.

[24] Blair RE, Sombati S, Lawrence DC, McCay BD, DeLorenzo RJ (2004). Epileptogenesis causes acute and chronic increases in GABAA receptor endocytosis that contributes to the induction and maintenance of seizures in the hippocampal culture model of acquired epilepsy. J. Pharmacol. Exp. Ther.

[25] Morris TA, Jafari N, Rice AC, Vasconcelos O, DeLorenzo RJ (1999). Persistent increased DNA-binding and expression of serum response factor occur with epilepsy-associated long-term plasticity changes. J. Neurosci.

[26] Brookes PS, Levonen AL, Shiva S, Sarti P, Darley-Usmar VM (2002). Mitochondria: regulators of signal transduction by reactive oxygen and nitrogen species. Free Radic. Biol. Med.

[27] McCormack JG, Denton RM (1993). Mitochondrial Ca2+ transport and the role of intramitochondrial Ca2+ in the regulation of energy metabolism. Dev. Neurosci.

[28] Perez-Campo R, Lopez-Torres M, Cadenas S, Rojas C, Barja G (1998). The rate of free radical production as a determinant of the rate of aging: evidence from the comparative approach. J. Comp. Physiol. B.

[29] Brookes PS, Yoon Y, Robotham JL, Anders MW, Sheu SS (2004). Calcium, ATP, and ROS: a mitochondrial love-hate triangle. Am. J. Physiol. Cell Physiol.

[30] Poon HF, Calabrese V, Scapagnini G, Butterfield DA (2004). Free radicals: key to brain aging and heme oxygenase as a cellular response to oxidative stress. J. Gerontol. A Biol. Sci. Med. Sci.

[31] Feissner RF, Skalska J, Gaum WE, Sheu SS (2009). Crosstalk signaling between mitochondrial Ca2+ and ROS. Front. Biosci.

[32] Halliwell B, Gutteridge J (2007). Free Radicals in Biology and Medicine.

[33] Zoratti M, Szabo I (1995). The mitochondrial permeability transition. Biochim. Biophys. Acta.

[34] Mahadik SP, Mukherjee S (1996). Free radical pathology and antioxidant defense in schizophrenia: a review. Schizophr. Res.

[35] Halliwell B (1992). Reactive oxygen species and the central nervous system. J. Neurochem.

[36] Halliwell B (2001). Role of free radicals in the neurodegenerative diseases: therapeutic implications for antioxidant treatment. Drugs Aging.

[37] Shivakumar BR, Anandatheerthavarada HK, Ravindranath V (1991). Free radical scavenging systems in developing rat brain. Int. J. Dev. Neurosci.

[38] Coyle JT, Puttfarcken P (1993). Oxidative stress, glutamate, and neurodegenerative disorders. Science.

[39] Sayre LM, Moreira PI, Smith MA, Perry G (2005). Metal ions and oxidative protein modification in neurological disease. Ann Ist Super Sanita.

[40] Facchinetti F, Dawson VL, Dawson TM (1998). Free radicals as mediators of neuronal injury. Cell Mol. Neurobiol.

[41] Nunomura A, Moreira PI, Takeda A, Smith MA, Perry G (2007). Oxidative RNA damage and neurodegeneration. Curr. Med. Chem.

[42] Zeviani M, Muntoni F, Savarese N, Serra G, Tiranti V, Carrara F, Mariotti C, DiDonato S (1993). A MERRF/MELAS overlap syndrome associated with a new point mutation in the mitochondrial DNA tRNA(Lys) gene. Eur. J. Hum. Genet.

[43] Shoffner JM, Lott MT, Lezza AM, Seibel P, Ballinger SW, Wallace DC (1990). Myoclonic epilepsy and ragged-red fiber disease (MERRF) is associated with a mitochondrial DNA tRNA(Lys) mutation. Cell.

[44] Goto Y, Nonaka I, Horai S (1991). A new mtDNA mutation associated with mitochondrial myopathy, encephalopathy, lactic acidosis and stroke-like episodes (MELAS). Biochim. Biophys. Acta.

[45] Canafoglia L, Franceschetti S, Antozzi C, Carrara F, Farina L, Granata T, Lamantea E, Savoiardo M, Uziel G, Villani F, Zeviani M, Avanzini G (2001). Epileptic phenotypes associated with mitochondrial disorders. Neurology.

[46] Poon HF, Calabrese V, Scapagnini G, Butterfield DA (2004). Free radicals and brain aging. Clin. Geriatr. Med.

[47] Chamoun R, Suki D, Gopinath SP, Goodman JC, Robertson C (2010). Role of extracellular glutamate measured by cerebral microdialysis in severe traumatic brain injury. J. Neurosurg.

[48] Hatefi Y (1985). The mitochondrial electron transport and oxidative phosphorylation system. Annu. Rev. Biochem.

[49] Kudin AP, Zsurka G, Elger CE, Kunz WS (2009). Mitochondrial involvement in temporal lobe epilepsy. Exp. Neurol.

[50] Kunz WS, Kudin AP, Vielhaber S, Blumcke I, Zuschratter W, Schramm J, Beck H, Elger CE (2000). Mitochondrial complex I deficiency in the epileptic focus of patients with temporal lobe epilepsy. Ann. Neurol.

[51] Lee YM, Kang HC, Lee JS, Kim SH, Kim EY, Lee SK, Slama A, Kim HD (2008). Mitochondrial respiratory chain defects: underlying etiology in various epileptic conditions. Epilepsia.

[52] Kudin AP, Kudina TA, Seyfried J, Vielhaber S, Beck H, Elger CE, Kunz WS (2002). Seizure-dependent modulation of mitochondrial oxidative phosphorylation in rat hippocampus. Eur. J. Neurosci.

[53] Chuang YC, Chang AY, Lin JW, Hsu SP, Chan SH (2004). Mitochondrial dysfunction and ultrastructural damage in the hippocampus during kainic acid-induced status epilepticus in the rat. Epilepsia.

[54] Sleven H, Gibbs JE, Heales S, Thom M, Cock HR (2006). Depletion of reduced glutathione precedes inactivation of mitochondrial enzymes following limbic status epilepticus in the rat hippocampus. Neurochem. Int.

[55] Fujikawa DG, Shinmei SS, Cai B (2000). Kainic acid-induced seizures produce necrotic, not apoptotic, neurons with internucleosomal DNA cleavage: implications for programmed cell death mechanisms. Neuroscience.

[56] Kudin AP, Bimpong-Buta NY, Vielhaber S, Elger CE, Kunz WS (2004). Characterization of superoxide-producing sites in isolated brain mitochondria. J. Biol. Chem.

[57] Navarro A (2004). Mitochondrial enzyme activities as biochemical markers of aging. Mol. Aspects Med.

[58] MacMillan-Crow LA, Crow JP, Thompson JA (1998). Peroxynitrite-mediated inactivation of manganese superoxide dismutase involves nitration and oxidation of critical tyrosine residues. Biochemistry.

[59] Hider RC, Ma Y, Molina-Holgado F, Gaeta A, Roy S (2008). Iron chelation as a potential therapy for neurodegenerative disease. Biochem. Soc. Trans.

[60] Pun PB, Lu J, Moochhala S (2009). Involvement of ROS in BBB dysfunction. Free Radic. Res.

[61] Crompton M, Ellinger H, Costi A (1988). Inhibition by cyclosporin A of a Ca2+-dependent pore in heart mitochondria activated by inorganic phosphate and oxidative stress. Biochem. J.

[62] Beutner G, Ruck A, Riede B, Welte W, Brdiczka D (1996). Complexes between kinases, mitochondrial porin and adenylate translocator in rat brain resemble the permeability transition pore. FEBS Lett.

[63] Loeffler M, Kroemer G (2000). The mitochondrion in cell death control: certainties and incognita. Exp. Cell Res.

[64] Crompton M (1999). The mitochondrial permeability transition pore and its role in cell death. Biochem. J.

[65] Urbanska EM, Blaszczak P, Saran T, Kleinrok Z, Turski WA (1998). Mitochondrial toxin 3-nitropropionic acid evokes seizures in mice. Eur. J. Pharmacol.

[66] Yamamoto H (1995). A hypothesis for cyanide-induced tonic seizures with supporting evidence. Toxicology.

[67] Kann O, Kovacs R, Njunting M, Behrens CJ, Otahal J, Lehmann TN, Gabriel S, Heinemann U (2005). Metabolic dysfunction during neuronal activation in the *ex vivo* hippocampus from chronic epileptic rats and humans. Brain.

[68] Kunz WS (2002). The role of mitochondria in epileptogenesis. Curr. Opin. Neurol.

[69] Jarrett SG, Liang LP, Hellier JL, Staley KJ, Patel M (2008). Mitochondrial DNA damage and impaired base excision repair during epileptogenesis. Neurobiol. Dis.

[70] Junker H, Spate K, Suofu Y, Walther R, Schwarz G, Kammer W, Nordheim A, Walker LC, Runge U, Kessler C, Popa-Wagner A (2005). Proteomic identification of the involvement of the mitochondrial rieske protein in epilepsy. Epilepsia.

[71] Margerison JH, Corsellis JA (1966). Epilepsy and the temporal lobes. A clinical, electroencephalographic and neuropathological study of the brain in epilepsy, with particular reference to the temporal lobes. Brain.

[72] Singh R, Pathak DN (1990). Lipid peroxidation and glutathione peroxidase, glutathione reductase, superoxide dismutase, catalase, and glucose-6-phosphate dehydrogenase activities in FeCl3-induced epileptogenic foci in the rat brain. Epilepsia.

[73] Bruce AJ, Baudry M (1995). Oxygen free radicals in rat limbic structures after kainate-induced seizures. Free Radic. Biol. Med.

[74] Aytan N, Jung T, Tamturk F, Grune T, Kartal-Ozer N (2008). Oxidative stress related changes in the brain of hypercholesterolemic rabbits. Biofactors.

[75] Lopez J, Gonzalez ME, Lorigados L, Morales L, Riveron G, Bauza JY (2007). Oxidative stress markers in surgically treated patients with refractory epilepsy. Clin. Biochem.

[76] Yis U, Seckin E, Kurul SH, Kuralay F, Dirik E (2009). Effects of epilepsy and valproic acid on oxidant status in children with idiopathic epilepsy. Epilepsy Res.

[77] Freitas RM (2009). Investigation of oxidative stress involvement in hippocampus in epilepsy model induced by pilocarpine. Neurosci. Lett.

[78] Turkdogan D, Toplan S, Karakoc Y (2002). Lipid peroxidation and antioxidative enzyme activities in childhood epilepsy. J. Child Neurol.

[79] Sudha K, Rao AV, Rao A (2001). Oxidative stress and antioxidants in epilepsy. Clin. Chim. Acta.

[80] Aycicek A, Iscan A (2007). The effects of carbamazepine, valproic acid and phenobarbital on the oxidative and antioxidative balance in epileptic children. Eur. Neurol.

[81] Naziroglu M, Kutluhan S, Yilmaz M (2008). Selenium and topiramate modulates brain microsomal oxidative stress values, Ca2+-ATPase activity, and EEG records in pentylentetrazol-induced seizures in rats. J Membr Biol.

[82] Gluck MR, Jayatilleke E, Shaw S, Rowan AJ, Haroutunian V (2000). CNS oxidative stress associated with the kainic acid rodent model of experimental epilepsy. Epilepsy Res.

[83] Verrotti A, Basciani F, Trotta D, Pomilio MP, Morgese G, Chiarelli F (2002). Serum copper, zinc, selenium, glutathione peroxidase and superoxide dismutase levels in epileptic children before and after 1 year of sodium valproate and carbamazepine therapy. Epilepsy Res.

[84] Gunes S, Dirik E, Yis U, Seckin E, Kuralay F, Kose S, Unalp A (2009). Oxidant status in children after febrile seizures. Pediatr. Neurol.

[85] Verrotti A, Scardapane A, Franzoni E, Manco R, Chiarelli F (2008). Increased oxidative stress in epileptic children treated with valproic acid. Epilepsy Res.

[86] Hamed SA, Abdellah MM, El-Melegy N (2004). Blood levels of trace elements, electrolytes, and oxidative stress/antioxidant systems in epileptic patients. J. Pharmacol. Sci.

[87] Solowiej E, Sobaniec W (2003). The effect of antiepileptic drug therapy on antioxidant enzyme activity and serum lipid peroxidation in young patients with epilepsy. Neurol. Neurochir. Pol.

[88] Liu CS, Wu HM, Kao SH, Wei YH (1998). Serum trace elements, glutathione, copper/zinc superoxide dismutase, and lipid peroxidation in epileptic patients with phenytoin or carbamazepine monotherapy. Clin. Neuropharmacol.

[89] Karikas GA, Schulpis KH, Bartzeliotou A, Regoutas S, Thanopoulou C, Papaevangelou V, Giannoulia-Karantana A, Papassotiriou I, Fytou-Pallikari A (2009). Early effects of sodium valproate monotherapy on serum paraoxonase/arylesterase activities. Scand J. Clin. Lab Invest.

[90] Schulpis KH, Lazaropoulou C, Regoutas S, Karikas GA, Margeli A, Tsakiris S, Papassotiriou I (2006). Valproic acid monotherapy induces DNA oxidative damage. Toxicology.

[91] Yuksel A, Cengiz M, Seven M, Ulutin T (2000). Erythrocyte glutathione, glutathione peroxidase, superoxide dismutase and serum lipid peroxidation in epileptic children with valproate and carbamazepine monotherapy. J. Basic Clin. Physiol. Pharmacol.

[92] Niketic V, Ristic S, Saicic ZS, Spasic M, Buzadzic B, Stojkovic M (1995). Activities of antioxidant enzymes and formation of the glutathione adduct of hemoglobin (Hb ASSG) in epileptic patients with long-term antiepileptic therapy. FARMACO.

[93] Sobaniec W, Solowiej E, Kulak W, Bockowski L, Smigielska-Kuzia J, Artemowicz B (2006). Evaluation of the influence of antiepileptic therapy on antioxidant enzyme activity and lipid peroxidation in erythrocytes of children with epilepsy. J. Child Neurol.

[94] Graf WD, Oleinik OE, Glauser TA, Maertens P, Eder DN, Pippenger CE (1998). Altered antioxidant enzyme activities in children with a serious adverse experience related to valproic acid therapy. Neuropediatrics.

[95] Michoulas A, Tong V, Teng XW, Chang TK, Abbott FS, Farrell K (2006). Oxidative stress in children receiving valproic acid. J. Pediatr.

[96] Varoglu AO, Yildirim A, Aygul R, Gundogdu OL, Sahin YN (2010). Effects of valproate, carbamazepine, and levetiracetam on the antioxidant and oxidant systems in epileptic patients and their clinical importance. Clin. Neuropharmacol.

[97] Mahle C, Dasgupta A (1997). Decreased total antioxidant capacity and elevated lipid hydroperoxide concentrations in sera of epileptic patients receiving phenytoin. Life Sci.

[98] Ono H, Sakamoto A, Sakura N (2000). Plasma total glutathione concentrations in epileptic patients taking anticonvulsants. Clin. Chim. Acta.

[99] Yuksel A, Cengiz M, Seven M, Ulutin T (2001). Changes in the antioxidant system in epileptic children receiving antiepileptic drugs: two-year prospective studies. J. Child Neurol.

[100] Cengiz M, Yuksel A, Seven M (2000). The effects of carbamazepine and valproic acid on the erythrocyte glutathione, glutathione peroxidase, superoxide dismutase and serum lipid peroxidation in epileptic children. Pharmacol. Res.

[101] Arhan E, Serdaroglu A, Ozturk B, Ozturk HS, Ozcelik A, Kurt N, Kutsal E, Sevinc N (2011). Effects of epilepsy and antiepileptic drugs on nitric oxide, lipid peroxidation and xanthine oxidase system in children with idiopathic epilepsy. Seizure.

[102] Kurekci AE, Alpay F, Tanindi S, Gokcay E, Ozcan O, Akin R, Isimer A, Sayal A (1995). Plasma trace element, plasma glutathione peroxidase, and superoxide dismutase levels in epileptic children receiving antiepileptic drug therapy. Epilepsia.

[103] Karabiber H, Yakinci C, Durmaz Y, Temel I, Mehmet N (2004). Serum nitrite and nitrate levels in epileptic children using valproic acid or carbamazepine. Brain Dev.

[104] Kupferberg H (2001). Animal models used in the screening of antiepileptic drugs. Epilepsia.

[105] Safar MM, Abdallah DM, Arafa NM, Abdel-Aziz MT (2010). Magnesium supplementation enhances the anticonvulsant potential of valproate in pentylenetetrazol-treated rats. Brain Res.

[106] Johannessen SI, Landmark CJ (2010). Antiepileptic drug interactions - principles and clinical implications. Curr. Neuropharmacol.

[107] Ashrafi MR, Shams S, Nouri M, Mohseni M, Shabanian R, Yekaninejad MS, Chegini N, Khodadad A, Safaralizadeh R (2007). A probable causative factor for an old problem: selenium and glutathione peroxidase appear to play important roles in epilepsy pathogenesis. Epilepsia.

[108] Ozden H, Kabay SC, Toker A, Ustuner MC, Ozbayer C, Ustuner D, Gunes HV (2010). The effects of levetiracetam on urinary 15f-2t-isoprostane levels in epileptic patients. Seizure.

[109] Bolayir E, Celik K, Tas A, Topaktas S, Bakir S (2004). The effects of oxcarbazepine on oxidative stress in epileptic patients. Methods Find Exp. Clin. Pharmacol.

[110] Reiter RJ, Tan DX, Sainz RM, Mayo JC, Lopez-Burillo S (2002). Melatonin: reducing the toxicity and increasing the efficacy of drugs. J. Pharm. Pharmacol.

[111] Loscher W, Schmidt D (2006). Experimental and clinical evidence for loss of effect (tolerance) during prolonged treatment with antiepileptic drugs. Epilepsia.

[112] Bjornsson E (2008). Hepatotoxicity associated with antiepileptic drugs. Acta Neurol. Scand.

[113] Lu W, Uetrecht JP (2008). Peroxidase-mediated bioactivation of hydroxylated metabolites of carbamazepine and phenytoin. Drug Metab. Dispos.

[114] Ezz HS, Khadrawy YA, Noor NA (2011). The neuroprotective effect of curcumin and Nigella sativa oil against oxidative stress in the pilocarpine model of epilepsy: a comparison with valproate. Neurochem. Res.

[115] Arora T, Mehta AK, Sharma KK, Mediratta PK, Banerjee BD, Garg GR, Sharma AK (2010). Effect of carbamazepine and lamotrigine on cognitive function and oxidative stress in brain during chemical epileptogenesis in rats. Basic Clin. Pharmacol. Toxicol.

[116] Jain S, Bharal N, Khurana S, Mediratta PK, Sharma KK (2011). Anticonvulsant and antioxidant actions of trimetazidine in pentylenetetrazole-induced kindling model in mice. Naunyn Schmiedebergs Arch. Pharmacol.

[117] Naziroglu M, Kutluhan S, Uguz AC, Celik O, Bal R, Butterworth PJ (2009). Topiramate and vitamin e modulate the electroencephalographic records, brain microsomal and blood antioxidant redox system in pentylentetrazol-induced seizure of rats. J. Membr. Biol.

[118] Imao K, Wang H, Komatsu M, Hiramatsu M (1998). Free radical scavenging activity of fermented papaya preparation and its effect on lipid peroxide level and superoxide dismutase activity in iron-induced epileptic foci of rats. Biochem. Mol. Biol. Int.

[119] Kutluhan S, Naziroglu M, Celik O, Yilmaz M (2009). Effects of selenium and topiramate on lipid peroxidation and antioxidant vitamin levels in blood of pentylentetrazol-induced epileptic rats. Biol. Trace Elem. Res.

[120] Agarwal NB, Agarwal NK, Mediratta PK, Sharma KK (2011). Effect of lamotrigine, oxcarbazepine and topiramate on cognitive functions and oxidative stress in PTZ-kindled mice. Seizure.

[121] Oliveira AA, Almeida JP, Freitas RM, Nascimento VS, Aguiar LM, Junior HV, Fonseca FN, Viana GS, Sousa FC, Fonteles MM (2007). Effects of levetiracetam in lipid peroxidation level, nitrite-nitrate formation and antioxidant enzymatic activity in mice brain after pilocarpine-induced seizures. Cell Mol. Neurobiol.

[122] Willmore LJ, Triggs WJ (1984). Effect of phenytoin and corticosteroids on seizures and lipid peroxidation in experimental posttraumatic epilepsy. J. Neurosurg.

[123] Mori A, Yokoi I, Noda Y, Willmore LJ (2004). Natural antioxidants may prevent posttraumatic epilepsy: a proposal based on experimental animal studies. Acta Med. Okayama.

[124] Komatsu M, Hiramatsu M, Willmore LJ (2000). Zonisamide reduces the increase in 8-hydroxy-2'-deoxyguanosine levels formed during iron-induced epileptogenesis in the brains of rats. Epilepsia.

[125] Rauca C, Zerbe R, Jantze H (1999). Formation of free hydroxyl radicals after pentylenetetrazol-induced seizure and kindling. Brain Res.

[126] Turski WA, Cavalheiro EA, Bortolotto ZA, Mello LM, Schwarz M, Turski L (1984). Seizures produced by pilocarpine in mice: a behavioral, electroencephalographic and morphological analysis. Brain Res.

[127] Fernandes MJ, Dube C, Boyet S, Marescaux C, Nehlig A (1999). Correlation between hypermetabolism and neuronal damage during status epilepticus induced by lithium and pilocarpine in immature and adult rats. J. Cereb. Blood Flow Metab.

[128] Bellissimo MI, Amado D, Abdalla DS, Ferreira EC, Cavalheiro EA, Naffah-Mazzacoratti MG (2001). Superoxide dismutase, glutathione peroxidase activities and the hydroperoxide concentration are modified in the hippocampus of epileptic rats. Epilepsy Res.

[129] Motte J, Fernandes MJ, Baram TZ, Nehlig A (1998). Spatial and temporal evolution of neuronal activation, stress and injury in lithium-pilocarpine seizures in adult rats. Brain Res.

[130] Pavone A, Cardile V (2003). An in vitro study of new antiepileptic drugs and astrocytes. Epilepsia.

[131] Barres BA (1991). Glial ion channels. Curr. Opin. Neurobiol.

[132] Porter JT, McCarthy KD (1997). Astrocytic neurotransmitter receptors in situ and in vivo. Prog Neurobiol.

[133] Cornell-Bell AH, Finkbeiner SM, Cooper MS, Smith SJ (1990). Glutamate induces calcium waves in cultured astrocytes: long-range glial signaling. Science.

[134] Cardile V, Pavone A, Renis M, Russo A, Perciavalle V (2000). Biological effects of tiagabine on primary cortical astrocyte cultures of rat. Neurosci. Lett.

[135] Cardile V, Pavone A, Renis M, Maci T, Perciavalle V (2001). Effects of Gabapentin and Topiramate in primary rat astrocyte cultures. Neuroreport.

[136] Wang JF, Azzam JE, Young LT (2003). Valproate inhibits oxidative damage to lipid and protein in primary cultured rat cerebrocortical cells. Neuroscience.

[137] Cui J, Shao L, Young LT, Wang JF (2007). Role of glutathione in neuroprotective effects of mood stabilizing drugs lithium and valproate. Neuroscience.

